# Lysine Acetylation and Succinylation in HeLa Cells and their Essential Roles in Response to UV-induced Stress

**DOI:** 10.1038/srep30212

**Published:** 2016-07-25

**Authors:** Hong Xu, Xuanyi Chen, Xiaoli Xu, Rongyi Shi, Shasha Suo, Kaiying Cheng, Zhiguo Zheng, Meixia Wang, Liangyan Wang, Ye Zhao, Bing Tian, Yuejin Hua

**Affiliations:** 1 Institute of Nuclear-Agricultural Sciences, Zhejiang University, Hangzhou, 310029, China; 2 Institute of Zhejiang Cancer Research, Zhejiang Cancer Hospital, Hangzhou, 310022, China; 3 Zhejiang Institute of Microbiology, Hangzhou, 310000, China

## Abstract

Lysine acetylation and succinylation are major types of protein acylation that are important in many cellular processes including gene transcription, cellular metabolism, DNA damage response. Malfunctions in these post-translational modifications are associated with genome instability and disease in higher organisms. In this study, we used high-resolution nano liquid chromatography-tandem mass spectrometry combined with affinity purification to quantify the dynamic changes of protein acetylation and succinylation in response to ultraviolet (UV)-induced cell stress. A total of 3345 acetylation sites in 1440 proteins and 567 succinylation sites in 246 proteins were identified, many of which have not been reported previously. Bioinformatics analysis revealed that these proteins are involved in many important biological processes, including cell signalling transduction, protein localization and cell metabolism. Crosstalk analysis between these two modifications indicated that modification switches might regulate protein function in response to UV-induced DNA damage. We further illustrated that FEN1 acetylation at different sites could lead to different cellular phenotypes, suggesting the multiple function involvement of FEN1 acetylation under DNA damage stress. These systematic analyses provided valuable resources and new insight into the potential role of lysine acetylation and succinylation under physiological and pathological conditions.

Throughout lifespan, a cell identifies and corrects damage to the DNA molecules that encode its genome. In human cells, both normal metabolic activities and environmental factors such as UV light and radiation can cause DNA damage, such as base modification, single- or double- strand breaks, and inter-strand cross-links^1^,^2^. The biological consequences of DNA damage depend on the type of lesions induced and its genomic localization. Eukaryotic DNA is packaged into chromatin that constitutes a physical barrier that prevents enzymes and regulatory factors from accessing DNA during replication, transcription, recombination and repair. DNA damage can block replication and transcription and this can ultimately lead to cell death. However, the efficiency of the DNA repair process controls the cellular response and determines cell fate, and the basic processes of DNA repair are highly conserved among prokaryotes and eukaryotes and even bacteriophage^3^. Our long-term goal is to decipher how cells process DNA lesions to maintain genomic integrity and post-translational modification (PTM) has emerged as an important factor. Reversible PTM of proteins plays a key role in regulating their activity, localization and interactions, all of which contribute to genome complexity and stability^4^. To date, more than 400 different types of PTM have been identified, including phosphorylation, methylation and ubiquitination and specific amino acid modifications can be used as biomarkers indicating DNA damage^5^.

The response to DNA damage involves the formation of protein complexes that sense DNA lesions and coordinate cell cycle progression, DNA repair, apoptosis and DNA damage tolerance pathways. PTM of histone and non-histone proteins is essential for the DNA damage repair machinery to assemble at sites of DNA damage^6^, but the exact mechanisms by which PTM participates in the temporal and spatial aspects of protein complex formation are poorly understood. Among the 20 amino acid residues in proteins, lysine is a frequent target for modification. Lysine acetylation has been extensively studied and is a important modification of proteins in eukaryotes and prokaryotes, influencing gene splicing, cell migration, cytoskeletal reorganization and DNA repair^7^,^8^. Studies of the acetylome have been reported in humans^9^, mice^10^, drosophila^11^, plants^12^, and bacteria^11^,^13^,^14^. Lysine acetylation is an evolutionarily conserved and widespread type of PTM that is functionally important and ubiquitous across all eukaryotes^15^, particularly in glycolysis, gluconeogenesis, the tricarboxylic acid (TCA) cycle, the urea cycle, fatty acid metabolism, and glycogen metabolism^16^,^17^.

Lysine succinylation was first reported in *E. coli*^18^ but has been observed in numerous organisms. Since lysine succinylation adds a larger structural moiety than acetylation or methylation, this modification induces greater changes in the chemical properties and protein structure and function^18^. Lysine acetylation and succinylation often target the same sites, and the dynamic changes in these two types of PTM in response to growth conditions or genetic mutations remain poorly characterized and understood.

Systematic comparison between lysine acetylation and succinylation in diverse organisms revealed extensive overlap^19^, raising the possibility of crosstalk. However, it remains to be determined whether different types of acylation have unique regulatory roles or perform redundant functions. The relative stoichiometry of different types of acylation occurring on the same lysine residues may also be important and worthy of study. In this study, we investigated the potential role of lysine acetylation and succinylation in the response to UV-induced DNA damage. Specifically, fundamental cellular processes involved in the detection and repair of DNA lesions were investigated, as was the activation of the DNA translesion synthesis- the DNA damage tolerance pathway, which is a major cause of drug resistance in tumours. This work therefore has relevance to public health for preventing and treating malignancies.

## Results

### Quantification of lysine-acetylated (Kac) and lysine-succinylated (Ksuc) peptides and proteins in HeLa cells following UV treatment

**Figure 1:**
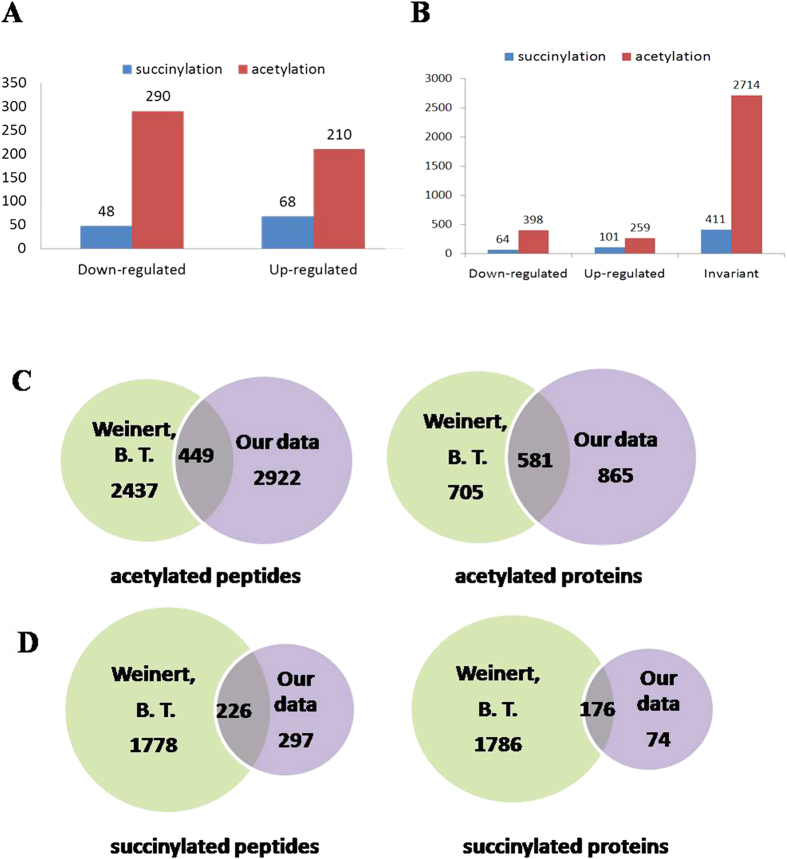
Quantitative overview of lysine-succinylated and lysine-acetylated peptides and proteins in *HeLa cells* following UV treatment. **(A)** Quantification of acetylated and succinylated proteins of HeLa cells in response to UV treatment. (**B**) Quantification of acetylated and succinylated peptides of HeLa cells in response to UV treatment. (**C**) Comparison of acetylated peptides and proteins identified in this study with previous work^15^. (**D**) Comparison of succinylated peptides and proteins identified in this study with previous work^15^.

Lysine succinylation was recently identified as a novel PTM, and has been shown to be extensive in both prokaryotes and eukaryotes^18^,^19^. Studies on succinylated and acetylated peptides and proteins in HeLa cells under normal growth conditions revealed that proteins involved in cellular metabolism (TCA cycle), fatty acids and porphyrin synthesis are subjected to this type of PTM. Additionally, numerous DNA repair proteins were identified to undergo lysine acetylation and succinylation, including FEN1, Rad23B, APEX1, and XRCC5, indicating a potential role in the response to and repair of cellular DNA damage, especially in the case of succinylation. To further evaluate the contribution of lysine acetylation and succinylation to DNA damage response, we pursued SILAC and bioinformatics analysis to compare the dynamic changes in acetylation (Sheet 1) and succinylation (Sheet 2) of proteins in HeLa cells under UV-induced stress. Quality control and validation of the MS data met all requirements (Supplementary Figure 1). MS data identified 3371 acetylation sites in 1446 proteins and 576 succinylation sites in 250 proteins, of which 3345 acetylation sites in 1440 proteins and 567 succinylation sites in 246 proteins were quantified. The modification level of Acetylated or succinylated proteins was considered to be up-regulated if greater than 1.5-fold higher, and down-regulated if less than 0.667-fold lower than controls. Based on this premise, 398 acetylation sites in 290 proteins were down-regulated and 259 acetylation sites in 210 proteins were up-regulated (Fig. 1A,B). Meanwhile, 102 succinylation sites in 68 proteins were up-regulated and 63 succinylation sites in 48 proteins were down-regulated in response to UV treatment in modificatino level(Fig. 1A,B).

Notably, compared with data published in 2013^19^, a greater number of acetylated peptides and proteins were identified in HeLa cells in this work. However, the coverage of the two datasets is relatively low (Fig. 1C, Sheet 3), which raises some questions concerning the sensitivity of the methods and the apparent dynamics of protein acetylation in response to different growth and stress conditions. Although less succinylated proteins were identified than previously reported, more than half of the quantified peptides were positively identified, and nearly 30% of proteins were novel (Fig. 1D, Sheet 4). Succinylation occurs at low levels, which brings technical challenges, and the data indicate that it occurred at many more sites than that were identified. The biological significance of both lysine acetylation and succinylation remain to be explored.

### Subcellular localization and functional annotation of changed Kac and Ksuc proteins

**Figure 2:**
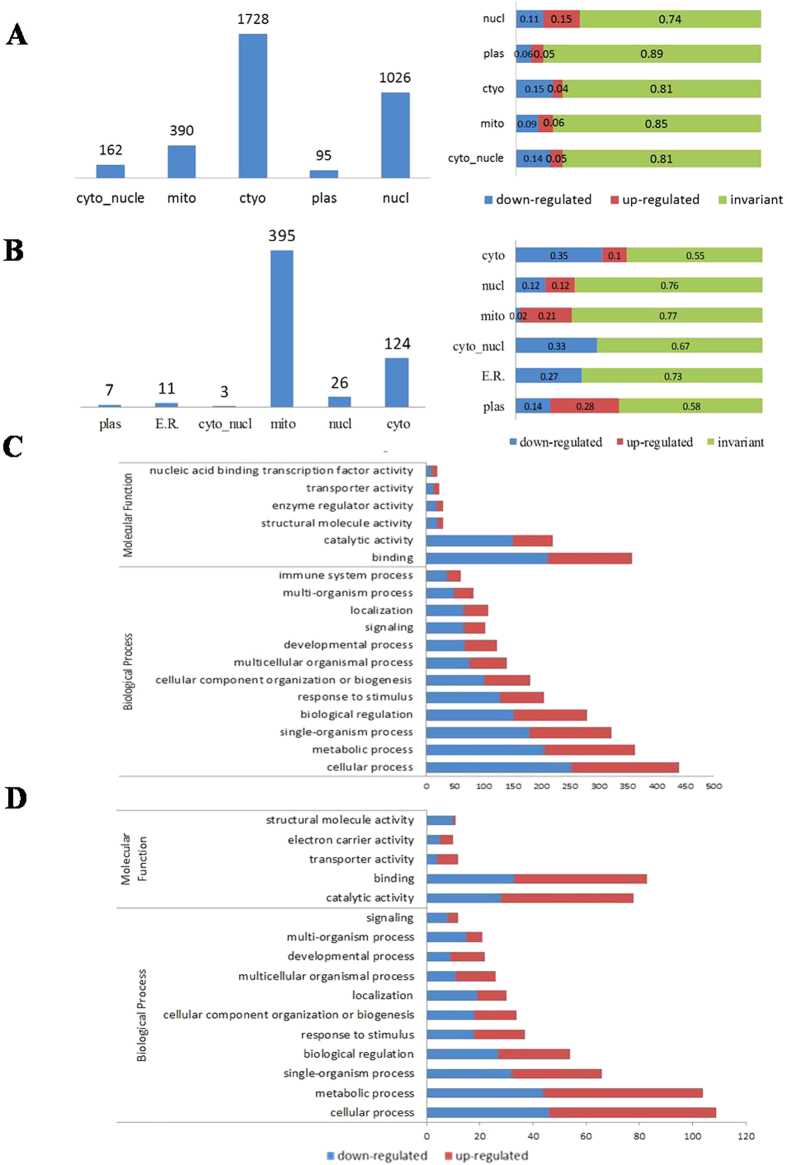
Subcellular localization and functional annotation of changed Kac and Ksuc proteins under UV treatment. (**A**) The distribution of lysine acetylation proteins. Most proteins locate in nuclear and cytoplasm. And the percentage of down-regulate proteins as well as up-regulated modification proteins are listed on the right histogram. (**B**) Subcellular distribution of succinylation proteins. A large number of them locate in mitochondria and cytoplasm. The histogram about the percentage of down-regulate proteins and up-regulated modification proteins is shown on the right. (**C**) GO analysis of acetylated proteins and the dynamic changing under UV treatment. (**D**) GO analysis of succinylated proteins and the dynamic changing under UV treatment.

We used WoLF PSORT software to predict the subcellular localization of quantified proteins under different conditions. Over half of all Kac proteins were distributed in the cytosol, and many others were present in the nucleus (Fig. 2A). The greatest changes in response to UV treatment were nuclear proteins. In modification level, approximately 15% of nuclear Kac proteins were up-regulated, and 11% were down-regulated. Meanwhile, 15% of cytosol Kac proteins and 14% of cytosolic_nuclear proteins were up-regulated, and 4% and 5% were down-regulated, respectively. Interestingly, about 70% of succinylated proteins were distributed in mitochondria, of which 21% were up-regulated under UV treatment (Fig. 2B). Many of the modified proteins were down-regulated (Fig. 2B), indicating the complexity of modification and relatively independent functions with acetylation.

To elucidate the potential role of lysine acetylation and succinylation in HeLa cells in response to UV damage, all identified proteins were submitted to GO functional classification analysis. A total of 1440 Kac proteins were annotated into two categories with numerous classifications. Most differentially abundant Kac proteins were associated with binding and catalytic activity, 147 and 212 binding-associated Kac proteins were up- and down-regulated in the modification level, respectively, and 69 and 151 catalytic activity-associated Kac-proteins were up- and down-regulated, respectively (Fig. 2C). These processes may therefore be regulated by PTM of lysine in HeLa cells after UV treatment. Acetylated variants were also closely connected with numerous biological progresses, including cellular process, metabolic process and single-organism process. A slightly higher proportion of these modified proteins were down-regulated, rather than up-regulated in the modification level (Fig. 2C). Among the succinylated proteins, many were associated with metabolic, cellular and single-organism processes (Fig. 2D). Lysine succinylation is believed to alter protein structure, suggesting changes in succinylation due to UV radiation could modify ligand binding and catalytic activity (Fig. 2D).

Following UV treatment, the levels of acetylated proteins involved in the Fanconi anaemia pathway DNA replication and mismatch repair were significantly increased (Sheet 5). The Fanconi anaemia pathway is required for replication fork stability under metabolic and DNA damage stress. Previous studies demonstrated that inter-strand cross-link (ICL) repair shares common proteins with mismatch repair^20^. Specifically, MutS complexes are involved in the recognition and early processing of ICLs. UBE2T is a critical E2 conjugating enzyme catalyzing FANCD2 mono ubiquitination in the FA pathway. UBE2T’s deficiency results in failure to excise DNA at sites near the ICLs^21^ and causes FA-T Subtype of Fanconi Anemia^22^. Still, UBE2T-mediated ubiquitin-signalling response pathway is proved to contribute to nucleotide excision repair^23^. In our study, the modified level of Replication protein A (RFA1), which participates in Fanconi anaemia and mismatch repair pathways as well as DNA replication, was up-regulated following UV treatment.

**Figure 3:**
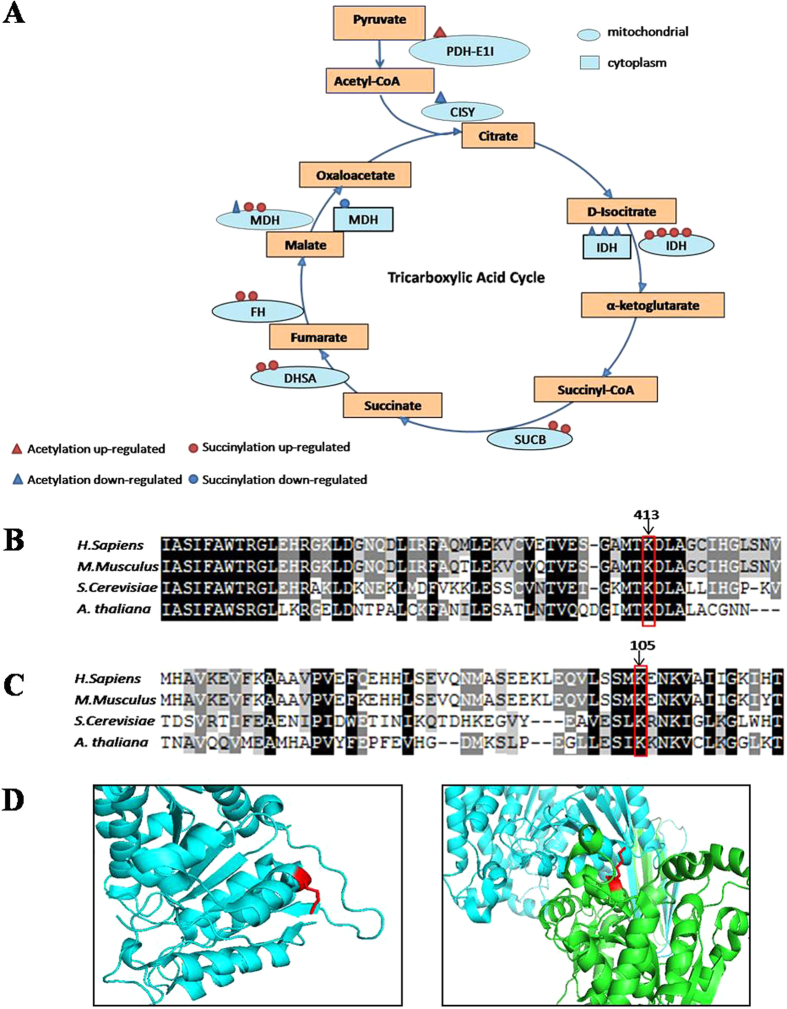
Bioinformatics analysis of enzymes involved in the tricarboxylic acid cycle. (**A**) The enzyme shown are Pyruvate dehydrogenase E1 component subunit alpha (PDH-E1I), Citrate synthase (CISY), isocitrate dehydrogenase (IDH), succinyl-CoA ligase subunit beta (SUCB), succinate dehydrogenase (DHSA), fumarate hydratase (FH), malate dehydrogenase (MDH). The triangle present acetylation sites and the circle present succinylation sites. Red present up-regulated modification and blue present the oppsite. The oval present mitochondrial and the rectangle present cytoplasm. (**B**) Clustal W alignment of isocitrate dehydrogenase NADP (mitochondrial) homologs from H. sapiens(GI:583966148), M. musculus (GI:225579033), S. cerevisiae (GI:124160), A. thaliana(GI:75246494). Conserved succinyllysine residues are labelled with arrows. (**C**) Isocitrate dehydrogenase NAD subunit beta (mitochondrial) homologs from H. sapiens (GI:385648280), M. musculus (GI:18700024), S. cerevisiae (GI:6324291), A. thaliana(GI:330251495). Conserved succinyllysine site are labelled with arrows. (**D**) The three-dimensional stucture were obtained from RCSB protein data bank. Succinylated and known functionally important sites are indicated by red sticks. Succinylated and known functionally important sites are indicated by red sticks. The positions are labled corresponding to the H. Sapiens sequence.

The TCA cycle is present in both the cytoplasm and mitochondria, and we identified a number of TCA enzymes for which lysine acetylation and succinylation level was up- or down-regulated (Fig. 3A). Proteins at all stages of the cycle were affected, suggesting that these PTMs influenced carbon metabolism following UV treatment. We performed sequence alignment to determine if succinylated sites were evolutionarily conserved among different species. Examination of four succinylation sites in isocitrate dehydrogenase identified two sequences that are conserved among *Homo sapiens*, *Mus musculus*, *Saccharomyces cerevisiae* and *Arabidopsis thaliana* (Fig. 3B,C). These conserved sequences were used to search the RCSB Protein Data Bank, and the mitochondrial isocitrate dehydrogenase NADP-binding subunit was identified. The conserved positively charged lysine (red in ) is located in an α-helix at the edge of the protein, and could therefore likely interact with negatively charged substrates such as proteins or nucleotides when succinylated. The same could be true for the isocitrate dehydrogenase NAD-binding beta subunit (Fig. 3D).

### Identification of Kac and Ksuc site motif and domain in response to UV treatment

**Figure 4:**
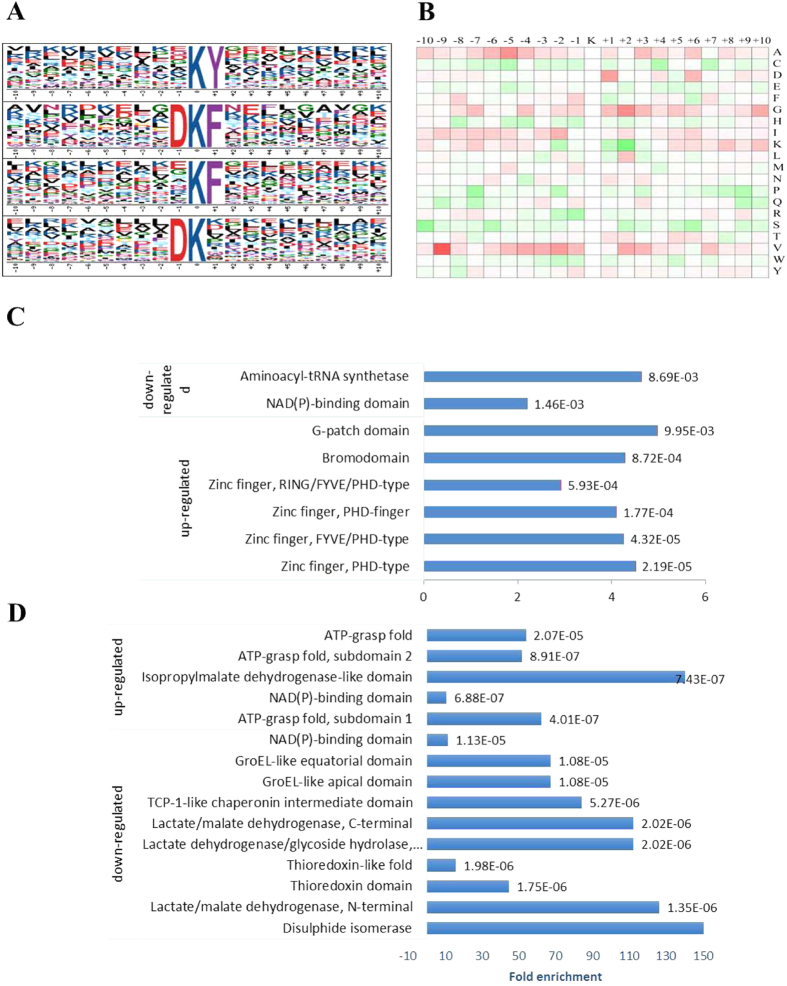
The specific motifs of Kac sites and Ksuc sites. (**A)** The specific motifs of acetylated lysine, aromatic amino acids like Y and F as well as a positively charged amino acid (H) were observed in the +1 position. And acidic amino acids (D) mostly presented in the −1 position. (**B**) The specific motifs of succinylated lysine. Hydrophobic amino acids like Alanine and valine present more than other amino acids surrounded lysine succinylation site. (**C**) Domain analysis of acetylated proteins. (**D**) Domain analysis of succinylated proteins.

To search for a possible consensus sequence motif around Kac sites, motif-x software was used to check acetyl-13-mer sequences (six amino acids upstream and downstream of the acetylation site). A total of six significantly enriched Kac site motifs were identified, among which an enrichment of aromatic amino acids (Y and F) and a positively charged amino acid (H) were observed in the +1 position, and an acidic amino acid (D) was generally present in the −1 position. KY or KH motifs have been found in *Drosophila melanogaster, Vibrio parahemolyticus, Streptomyces roseosporus, Bombyx mori* and *E. coli*, suggesting that aromatic and positively charged amino acids at these positions may be functionally important for acetylation. Positively charged amino acids were almost entirely absent from sites surrounding the acetylated lysine (Fig. 4A). Lysine succinylation motifs appear to be less specific than lysine acetylation sites, although this may be partly due to less sites being identified. The hydrophobic alanine and valine are particularly abundant at sites surrounding lysine succinylation sites (Fig. 4B).

The cellular localizations and function of proteins are often indicated by their domains. Acetylation sites are relatively abundant in bromodomains, G-patch domains and other Zinc finger-type domains (P < 0.01), indicating a role in DNA binding, which may explain their up-regulation. The Acetylation level of these sites are down-regulated in aminoacyl-tRNA synthetases and NAD(P)-binding domains (Fig. 4C). In contrast, succinylation sites appear to be more abundant in proteins associated with cellular metabolic process (Fig. 4D).

### Crosstalk between acetylation and succinylation proteins under UV-induced stress

**Figure 5:**
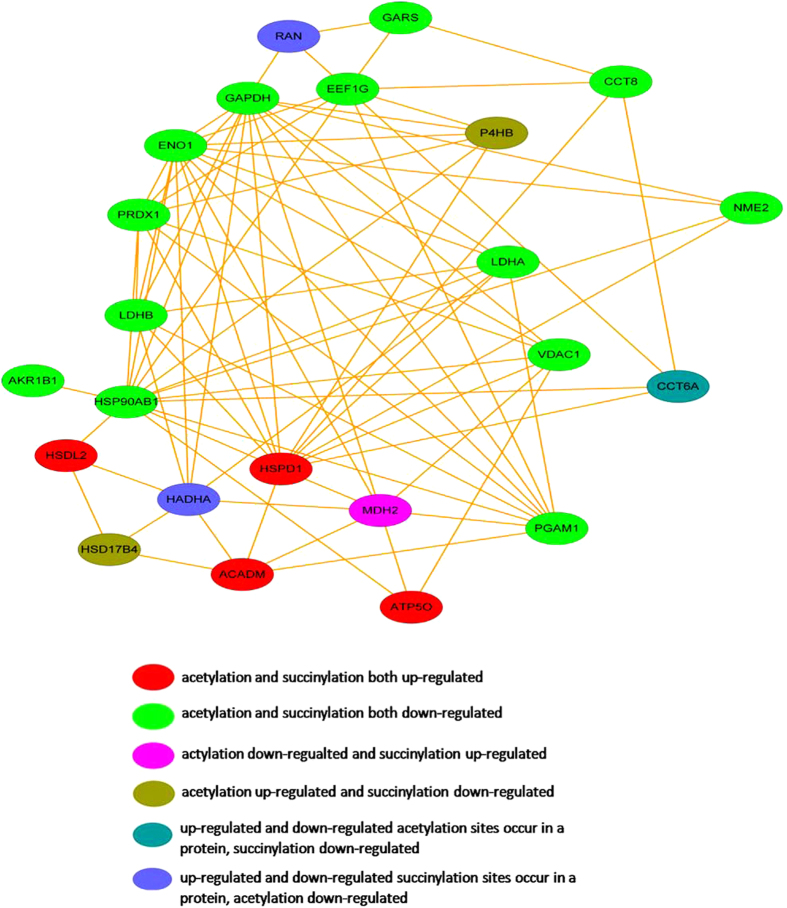
The crosstalk between acetylated and succinylated proteins under UV treatment. The red present acetylation and succinylation both up-regulated, the light green present acetylation and succinylation levels both down-regulated, the rose red present acetylation down-regulated and succinylation up-regulated, the dark green present acetylation up-regulated and succinylation down-regulated.

Previous studies provided a systems-wide view of succinylation and revealed an extensive overlap with acetylation, raising the possibility of crosstalk between these PTMs. However, it remains to be determined whether different types of acylation have unique regulatory roles or perform redundant functions. Also, it would be interesting for future studies to investigate the relative stoichiometry of different acylation types occurring on the same lysine residues. This research identified 576 succinylation sites on 250 proteins and 3371 acetylation sites on 1446 proteins, among which 27 proteins contained both acetylation and succinylation sites. In order to investigate the interaction between these two PTMs, we used the protein network interaction database STRING (version 10) to generate a protein network with a confidence level of 0.7. We then used CytoScape software to visually edit and analyze the network, with different colours for up-regulated and down-regulated levels of acetylation or succinylation sites (Fig. 5). Proteins were divided into six groups according to variations following UV treatment. Both types of PTMs were down-regulated for most proteins, and MDH2 was the only protein with down-regulated acetylation versus up-regulated succinylation. We verified the total acetylation and succinylation level of MDH2 before and after UV treatment, and found that acetylation level of MDH2 didn’t change too much but the succinylation level of MDH2 increased dramatically after treatment (Supplementary Figure 2), indicating the differential role of these two modification of MDH2. MDH2 is an important enzyme in carbon metabolism, and whether the succinylation and acetylation sites interact or influence protein function requires further investigation.

### Novel acetylation sites of FEN1 contribute to different cellular processes

**Figure 6:**
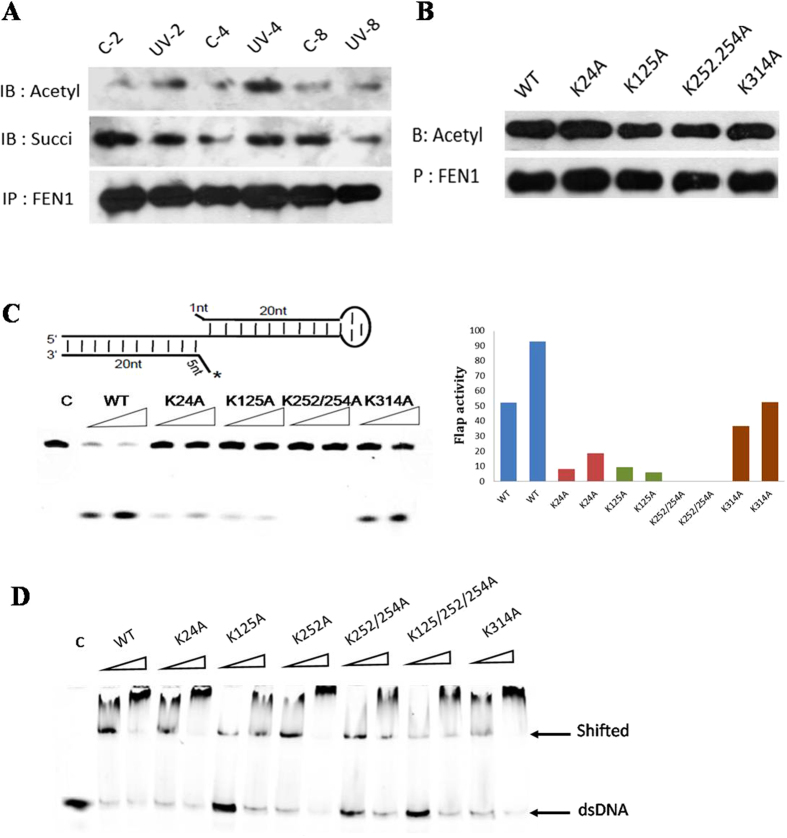
Validation the essential role of FEN1 acetylation in response to UV-induced cellular stress. (**A**) FEN1 acetylation and succinylation level after UV treatment. HeLa cells were transfected with 3XFlag tagged wildtype FEN1 (WT). After UV treatment, FEN1 was pulled down using M2 beads and the modification level was determined by western blotting. (**B**) Validation of FEN1 acetylation site *in vivo*. Each identified acetylation site was mutated into alanine, the plasmid was transfected into HeLa cells respectively. FEN1 was pulled down using M2 Beads and the acetylation level was determined by anti-acetylation antibody. (**C**) Cleavage activities of WT and mutant FEN1 proteins. Double flap DNA substrate labeled with FAM was annealed and incubated with 2 μM recombinant proteins for 2 or 5 minutes. The product was resolved using 15% denaturing PAGE, and visualized by autoradiography. (**D**) DNA binding activity of WT and mutant proteins. Double flap DNA (500 nM) incubated with different concentrations (80 or 160 nM) of purified proteins at 4 °C for 30 min and the products were loaded in Native PAGE. The gel was visualized by Typhoon 9500 and quantified by ImageJ.

**Figure 7:**
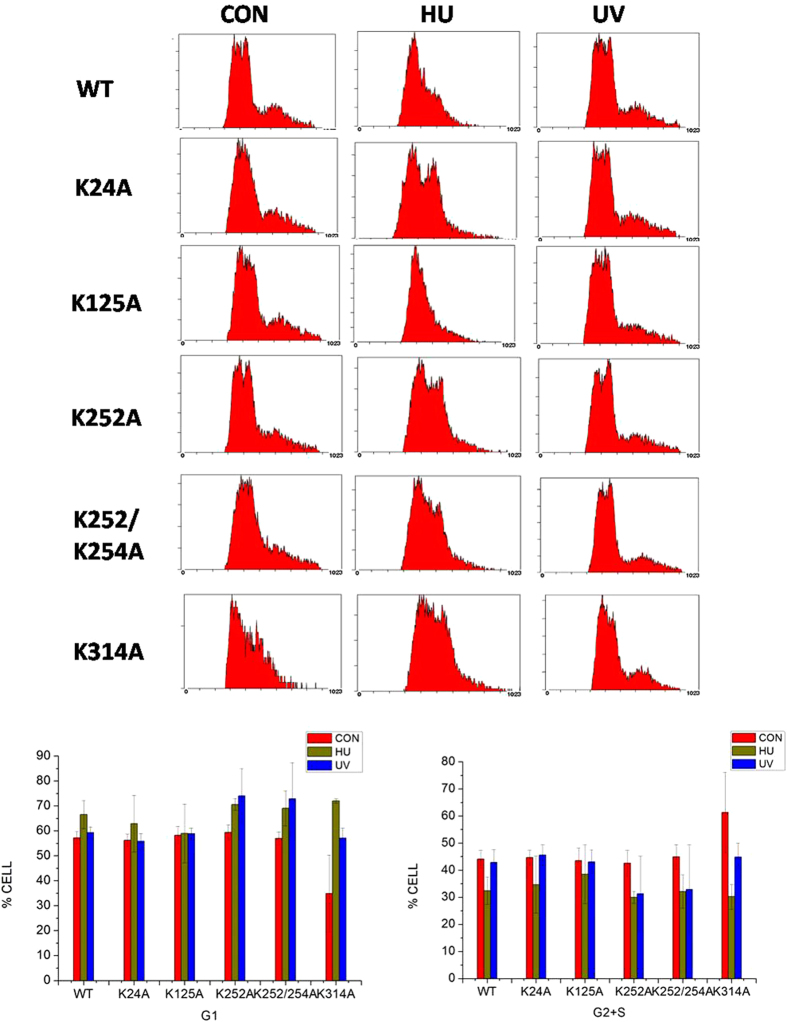
Cell cycle histograms of the different FEN1 overexpression cell lines in response to UV or HU treatment. HeLa cells were overexpressed with WT or FEN1 mutant (K24A, K125A, K252A, K252/K254A and K314A) and prepared for FACS analysis after HU or UV exposure as described in Materials and Methods. The number of cells in each phase of the cell cycle was obtained by using MultiCycle AV DNA Analysis software. The percentage of cells in each phase was shown below.

**Figure 8:**
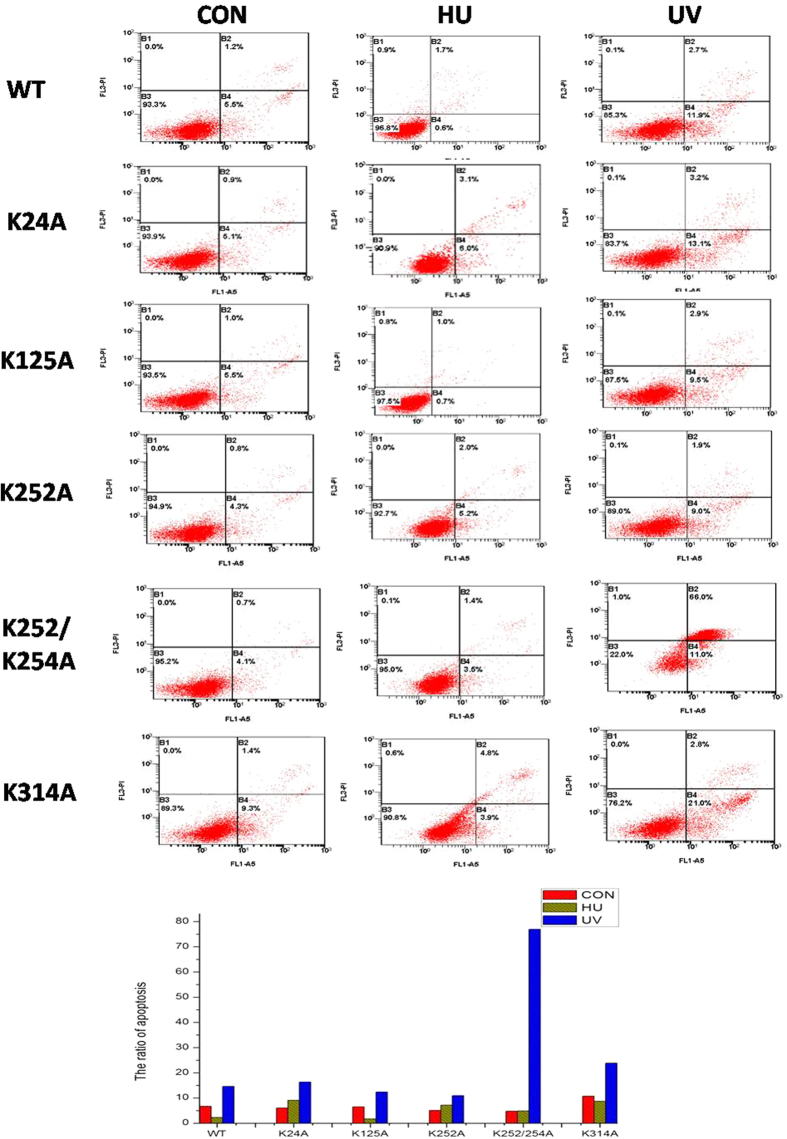
Flow cytometry analysis of UV or HU induced apoptosis in different FEN1 overexpression cells. Representative images of cell apoptosis in different HeLa cells with HU or UV treatments analyzed by flow cytometry using Annexin V FITC/PI double staining. Statistical analysis of the proportions of HeLa cells corresponding to early and late apoptotic cells.

In addition to the proteins involved in metabolism, numerous DNA repair proteins were also differentially acetylated or succinylated in response to UV treatment, including MSH2, XRCC5, and XRCC6. Some of the identified sites have not been reported previously such as the ones on FEN1. FEN1 mainly possessed three different nuclease activities based on the structure of DNA substrates^24^, and more than 10 PTM sites have now been identified from the present and previous reported studies^25^,^26^, most likely involved in protein binding, enzyme activity and protein-protein interactions, based on the three dimensional structure (Supplementary Figure 3A). FEN1 acetylation is known to affect its enzyme activity^27^, and PTM of this protein was altered markedly in response to UV damage^28^. In the present study, both acetylated and succinylated FEN1 levels were altered following UV treatment (Fig. 6A). The five acetylation sites (K24, K125, K252, K254, K314) identified in this study (Supplementary Figure 4) are conserved in eukaryotes, especially higher organisms (Supplementary Figure 3B). Mutation of these lysine residues to alanine was performed for *in vivo* verification, and the levels of acetylated K125A and K252\K254A decreased more than the other mutants (Fig. 6B). We purified these mutants using an *E. coli* expression system (Supplementary Figure 5A), and found that the flap activity of the K125A and K252\K254A mutants markedly decreased than that of others, 5~10% and almost zero respectively compared with the wild type protein(50~90%) (Fig. 6C). This endonuclease activity deficiency was partially due to the decrease of their DNA binding activity which K125 mutant’s binding activity is less than 10% and K252\K254A mutant’s binding activity is 20%, while the activity of wide type protein is nearly 90% (Fig. 6D, Supplementary Figure 5B,C). To further verify the biological functions of FEN1 with acetylation at the five diffrent sites, we used flow cytometry to analyse the cell cycle progression and apoptosis changes under UV- and Hydroxyurea (HU)-induced stress in different FEN1 mutants’ transfected cells (Supplementary Figure 6). After treatment, the percentage of HeLa cells with WT overexperssion in G1 phase increased about 10%, but the ratio of cells contain K125A mutant didn’t show too much changes after treatment, indicating a deficient cell cycle arrest in response to cellular stresses (Fig. 7). Meanwhile, the cells with K252A or K252/K254A mutant have much higher G1 phase ratio 70%, increase almost 10% compared to others, also suggesting their important role in cell cycle. We also tested the apoptosis ratio of those cells under two treatment, and found that K252/K254A mutant cells were much more sensitive to UV induced DNA damage and showed much higher percentage(75%) of apoptosis cells compared with others(10–20%) (Fig. 8). Interestingly, K314A mutant overexpression cells show different cell cycle pattern and have slightly higher sensitivity to UV treatment. All of these indicating that FEN1 acetylation at different sites are closely related to genome instability through different cellular processes,acetylation and succinylation of FEN1 have different biological rolesto regulate its function under different conditions. Systematic study in the future will help us to demonstrate the underlying mechanism of this well organized regulation.

## Discussion

In present work, we investigated global changes in lysine acetylation and succinylation in HeLa cells following UV-induced stress. We utilized a standardized workflow for acetylome analysis and identified 3371 acetylated and 523 succinylated peptides. Comparing with a previously reported dataset, less than 25% acetylated and 50% succinylated peptides were confirmed in their study. This relatively low coverage may due to the following reasons. First, the experimental procedure was different in two studies. HPLC fractionation was performed before anti-acetyllysin enrichment in this study while the Weinert *et al*. enriched the acetyllysin peptides before fractionation. In addition, the difference of anti-acetyllysin antibodies used in the two researches may result in the relatively low overlap rate. Besides, different amount of initial total protein were used for succinylated peptide enrichment in the two studies, which may be the main reason that more sucinylation peptides were identified in the Weinert study. Further optimization in the experimental settings would help to identify more protein for succinylome analysis in further.

Protein succinylation appeared to be more closely associated with cellular metabolism which mainly occurred in mitochondria, while acetylated proteins were mostly identified in cytosol, suggesting that the biological events could be quite different even the chemical properties was quite similar between those two modifications. The biological significance and mechanisms of succinylation and acetylation and further their crosstalk remain unknown. Our findings showed that MDH2 had a markedly increased level in succinylation modification under UV treatment, suggesting a functional switch of MDH2 through crosstalk or changed level of PTM modifications. Since many essential proteins participate in both regular housekeeping activities and DNA damage repair, PTMs may be required for switching their roles in response to stimuli.

Besides of the global identification, we also verified novel acetylation sites of FEN1 which is a very important protein in both DNA replication and repair. We constructed the mutations of these sites and found its enzyme activity and DNA binding activity could be changed in different levels. Cell cycle and apoptosis analysis showed that different mutants have different cellular phenotypes even the *in vitro* activity pattern looks similar. Since FEN1 protein interacted with various proteins and involved in multiple pathways, how these modifications affect its biological role and the underlying mechanisms are worthy of further invastigation. Our studies, together with previous findings, suggest that FEN1 succinylation and acetylation are under dynamic control in different conditions, dysregulation of its function lead to genome instability which is related to cell destiny. The mechanisms of how PTMs alter the function of proteins and hence influence genome stability and cell survival can be investigated using time-course-dependent proteomics experiments, including the whole proteome. And the set by set studies of post translational modifications will become very powerful tools for systematic functional analysis of targeted proteins.

## Materials and Methods

### Mass spectrometry sample preparation

HeLa cells were grown in Dulbecco’s modified Eagle’s medium (Dulbecco’s Modified Eagle Medium) supplemented with 10% fetal bovine serum and at 37 °C with 95% air and 5% CO_2_. Cells were labelled with heavy isotopic lysine (K6) and light isotopic lysine (K0) using a stable isotope labelling by/with amino acids in cell culture (SILAC) Protein Quantitation Kit (Thermo Fisher Scientific, San Jose, CA, USA) according to manufacturer’s instructions. Heavy and light forms were grown for more than six generations before harvesting to achieve more than 97% labelling efficiency. Cells were then further propagated in SILAC media to the desired cell density (~5 × 10^8^) in fifteen 150 cm^2^ flasks. K0 cells were treated with UV radiation (120 J/m^2^) and cultured for another 1 h, then harvested and washed twice with ice-cold PBS. After snap freezing in liquid nitrogen, cell pellets were stored at −80 °C until needed.

Harvested K0 and K6 labelled cells were sonicated three times on ice using a high intensity ultrasonic processor (Scientz, China) in lysis buffer (8 M urea, 10 mM DTT, 1% protease inhibitor cocktail III (Sigma USA)). The remaining debris was removed by centrifugation at 20,000 × *g* for 10 min at 4 °C. Then we applied 2-D quant method for protein concentration determination. After measuring the protein concentration, equal amounts of crude protein from K0 and K6 samples were precipitated by adding TCA to a final concentration of 15% (v/v) (soluble fraction). After washing twice with acetone at −20 °C, protein pellets were dissolved in 100 mM NH_4_HCO_3_ (pH 8.0) for trypsin digestion. Trypsin (Promega, Madison, WI) was added to the protein solution at a ratio of 1:50 (w/w) for digestion at 37 °C for 16 h. DTT was then added to a final concentration of 5 mM followed by incubation at 50 °C for 30 min. Iodoacetamide (IAA, Sigma, USA) was then added at a final concentration of 15 mM to alkylate proteins, and samples were incubated at room temperature in the dark for 30 min. The alkylation reaction was quenched with 30 mM cysteine at room temperature for another 30 min. Additional trypsin was then added at a ratio of 1:100 (w/w) for digestion at 37 °C for 4 h.

### Immunoaffinity enrichment of lysine-acetylated and lysine-succinylated peptides

All samples were fractionated by high pH reverse-phase HPLC using an Agilent(USA) 300 Extend C18 column (5 μm particles, 4.6 mm ID, 250 mm length). Briefly, peptides were initially separated with a gradient of 2% to 60% acetonitrile in 10 mM ammonium bicarbonate (pH 10) over 80 min into 80 fractions, and peptides were combined into 12 fractions and dried by vacuum centrifuge.

Succinylated or acetylated lysine peptides were enriched by the immunoaffinity procedure as previously described^22^. In brief, dried digests were redissolved in NETN buffer (100 mM NaCl, 1 mM EDTA, 50 mM Tris–HCl pH 8.0, 0.5% Nonidet P-40) and incubated with anti-succinyllysine or anti-acetyllysine agarose beads (PTM Biolabs) at a ratio of 20 μl of beads per mg of protein at 4 °C overnight with gentle rotation. After incubation, the supernatant was removed, and beads were carefully washed three times with NETN buffer, twice with NET buffer (100 mM NaCl and 1 mM EDTA, 50 mM Tris–Cl, pH 8.0), and once with water. Bound peptides were eluted from the beads with 1% trifluoroacetic acid and dried in a SpeedVac. Peptides were desalted with C18 Zip Tips (Millipore, Billerica, MA, USA) according to the manufacturer’s instructions and subjected to HPLC-MS/MS analysis.

### Mass spectrometry and LC-MS/MS analysis

Two analyses were performed for each fraction. Half of each sample was dissolved in solvent A (0.1% Formic Acid (FA) in 2% acetonitrile (ACN)), directly loaded onto a reversed-phase pre-column (Acclaim PepMap 100, Thermo Fisher Scientific, San Jose, CA, USA) and peptide separation was performed using a reversed-phase analytical column (Acclaim PepMap RSLC, Thermo Scientific). Elution comprised an increase from 7% to 22% solvent B (0.1% FA in 98% ACN) for 24 min, 22% to 35% for 10 min, 35% to 80% over 5 min, and 80% for the last 3 min, all at a constant flow rate of 280 nl/min. Separation was carried out on an EASY-nLC 1000 UPLC (Ultra Performance Liquid Chromatography) system, and the resulting peptides were analyzed by a Q Exactive Plus hybrid quadrupole-Orbitrap mass spectrometer (Thermo Fisher Scientific, San Jose, CA).

Peptides were subjected to a nanospray ionization (NSI) source followed by tandem mass spectrometry (MS/MS) using a Q Exactive Plus (Thermo Fisher Scientific, San Jose, CA) coupled online to a UPLC. Intact peptides were detected in the Orbitrap at a resolution of 70,000. Peptides were selected for MS/MS using a normalized collision setting (NCE) setting of 28, and ion fragments were detected in the Orbitrap at a resolution of 17,500. A data-dependent procedure that alternated between one MS scan followed by 20 MS/MS scans was applied for the top 20 precursor ions above a threshold ion count of 2 × 10^4^ in the MS survey scan with 10 s dynamic exclusion. The electrospray voltage applied was 2.0 kV. Automatic gain control (AGC) was used to prevent overfilling of the ion trap, and 5 × 10^4^ ions were accumulated for generation of MS/MS spectra. For MS scans, the m/z scan range was 350 to 1800.

### Database searching

All raw data files obtained from mass spectrometry analysis were processed using MaxQuant software (version1.4.1.2, http://www.coxdocs.org/doku.php?id=maxquant:start). The mass spectra were compared against the *SwissprotHuman* (20,203 sequences) database and concatenated with the reverse decoy database (http://www.uniprot.org/uniprot/?query=organism:9606+reviewed:yes). Trypsin/P was specified as a cleavage enzyme, and the search allowed up to three missed cleavages, five charges, and five modifications per peptide. The Mass Error was set to 20 ppm for the first search, 5 ppm for the main search, and 0.02 Da for fragment ions. The mass error was set to 5 ppm for precursor ions and 0.02 Da for fragment ions. Carbamido-methylation of Cys was specified as a fixed modification, and oxidation on Met, succinylation on lysine (lysine + 100.01604), and acetylation of the protein N-terminus were specified as variable modifications. The false discovery rate thresholds for proteins, peptides, and modification sites were specified as 1%. Minimum peptide length was set at 7 animo acids. The site localization probability was set as >0.75.

### Bioinformatics analysis of lysine-acetylated and lysine-succinylated peptides and proteins

Bioinformatics analysis of the identified peptides and proteins was performed described previously^23^. Briefly, Gene Ontology (GO) annotation was performed using the UniProt-GOA database (http://www.ebi.ac.uk/GOA). The Kyoto Encyclopedia of Genes and Genomes (KEGG) pathway was annotated using the online KEGG Automatic Annotation Fisher’s exact test (two-tailed) was used to assess GO/KEGG/Pfam domain enrichment analysis. Amino acid sequence motifs (10 amino acids upstream and downstream of the modified lysine were analyzed using motif-X. Motif-based clustering analyses were also performed, and cluster membership was visualized using a heat map. Crosstalk between lysine acetylation and succinylation under UV stress was assessed using functional protein association networks visualized using the STRING database (version 10, http://string-db.org/), with a high confidence threshold of 0.7.

### Protein expression and purification

Purified human wild-type and mutant flap endonuclease nuclease were prepared as previously described^29^,^30^. Briefly, target genes were cloned into expression vector pET28b, and transformed into *Escherichia coli* strain BL21 (DE3). Cells were cultured and expression was induced with isopropyl β-D-1-thiogalactopyranoside. Collected cells were extracted and purified using the PrepEase high specificity Histidine tagged Protein Purification Kit (USB, Cleveland, OH, USA). Eluted proteins were concentrated in dialysis buffer (50% glycerol, 2 mM DTT, 20 mM Tris–HCl pH 8.0, 1 mM MgCl_2_, 20 mM NaCl, 0.02% NaN_3_). Protein concentration was determined using the Bio-Rad (Bradford) protein assay (Bio-Rad, Hercules, CA, USA) and protein purity was evaluated by SDS-PAGE.

### Nuclease activity assay

DNA substrates were labelled with FAM as indicated. Purified wildtype (WT) or mutant FEN1 protein was incubated at 37 °C with 1 pmol DNA substrate for the specified times in reaction buffer containing 50 mM Tris-HCl pH 7.5, 10 mM MgCl_2_, 1 mM DTT and 0.2 mg/ml bovine serum albumin. Reactions were stopped by addition of 20 μl gel loading buffer (90% formamide dye, 3 M EDTA, 0.02% bromophenol blue and 0.02% xylene cyanol), resolved using 15% denaturing PAGE, and visualized using autoradiography.

### Electrophoretic mobility shift assay

All the oligonucleotides were purchased and synthesized by Sangon (Shanghai). The oligonucleotides were 5-end labeled using 6-FAM. The DNA substrates used for binding in this study are shown in supplemental Fig. 4B. The annealing of double flap DNA as follows: heating at 95 °C for 10 min following by slow cooling to room temperature. DNA binding assays were performed by incubating Double flap DNA(500 nM) with different concentrations (80 or 160 nM) of purified protein WT and mutants in binding buffer (5 mM MES-KOH at pH 6.5, 50 mM KCl, 1 mg/ml BSA, 5 mM EDTA, 1 mM DTT) at 4 °C for 30 min. The reaction mixtures were then loaded to electrophoresis on 20% native polyacrylamide gel. The gel was scanned using Typhoon 9500 and then analysed by ImageJ Software. Data are means of three independent measurements; error bars indicate standard deviations.

### Cell transfection and western blotting

HeLa cells were grown in Dulbecco’s Modified Eagle Medium supplemented with 10% fetal bovine serum. Plasmids (PEZ vector with 3 × FLAG tagged cloned in different FEN1 protein sequences) were delivered into cells individually after the density reached 80%. After a 4–6 h incubation, media was replaced with normal culture medium and cells were collected after 24–48 h. Collected cells were lysed with RIPA buffer containing protease inhibitor cocktails (Roche, Basel, CH) and M2 Beads were added to collect the flag-tagged FEN1 proteins. Samples were denatured, separated by SDS-PAGE, transferred onto a PVDF membrane, and incubated with primary antibody (1000 × diluted, Abcam, Cambridge, GB) and secondary antibody (2000 × diluted, Ptg lab, Chicago, USA) sequentially after blocking. The membrane was further washed with PBST and developed using ECL reagents (Pierce).

### Flow cytometry assays

In cell cycle assay, A total of 1 × 10^6^ cells were seeded in each six-well plate and further incubated for 12 hrs to reach 50% confluent. Then the cells were transfected with 3xflag-FEN1-WT or 3xflag-FEN1 mutants by using TG-transfection (Gene Technologies). Cells were treated with 1 mM HU ((Hydroxyurea) or ddH_2_O 24 hrs later and harvested after 12 hrs. For UV treatment, cells were exposed to UV at 120 J/m^2^ and collected after 2 hrs post-incubation. Cell cycle assay and apoptosis assays were conducted using Annexin V FITC/PI apoptosis and cell cycle kit (MultiSciences70-APCC101-100), samples were run on Beckman CoulterFC500 MPL and the cell cycle data were analyzed using MultiCycle AV DNA Analysis software.

## Additional Information

**How to cite this article**: Xu, H. *et al*. Lysine Acetylation and Succinylation in HeLa Cells and their Essential Roles in Response to UV-induced Stress. *Sci. Rep.* **6**, 30212; doi: 10.1038/srep30212 (2016).

## Supplementary Material

Supplementary Information
