# Corrigendum: Lysine Acetylation and Succinylation in HeLa Cells and their Essential Roles in Response to UV-induced Stress

**DOI:** 10.1038/srep46831

**Published:** 2017-05-26

**Authors:** Hong Xu, Xuanyi Chen, Xiaoli Xu, Rongyi Shi, Shasha Suo, Kaiying Cheng, Zhiguo Zheng, Meixia Wang, Liangyan Wang, Ye Zhao, Bing Tian, Yuejin Hua

Scientific Reports 6: Article number: 30212; 10.1038/srep30212 published online: 07 25 2016; updated: 05 26 2017.

In the original version of this Article, Supplementary Datasets 1, 2, 3, 4 and 5 were omitted. The correct Supplementary Datasets now accompany the Article.

