# Supplementary material for: Lysine Acetylation and Succinylation in HeLa Cells and their Essential Roles in Response to UV-induced Stress
Source: Sci Rep. 2016 Jul 25;6:30212. doi: 10.1038/srep30212 (PMC4959001; doi:10.1038/srep30212)
Supplement: Supplementary Information [file srep30212-s1.doc]

##### Identification of Novel Lysine Acetylation and Succinylation Sites in HeLa Cells and the Essential Role in Response to UV-induced Stress

##### Hong Xu^1, #^, Xuanyi Chen^1, #^, Xiaoli Xu^1, #^, Rongyi Shi^1^, Shasha Suo^1^, Kaiying Cheng^1^, Zhiguo Zheng^2^, Meixia Wang^3^, Liangyan Wang^1^, Ye Zhao^1^, Bing Tian^1^, Yuejin Hua^1, *^

**Supplementary Information (Tables and Figures)**


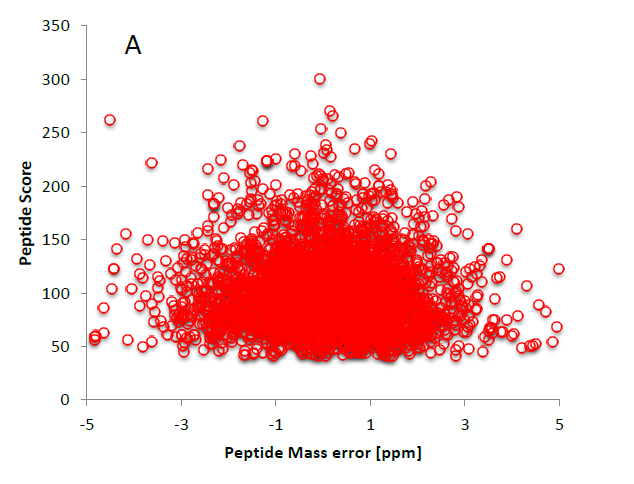

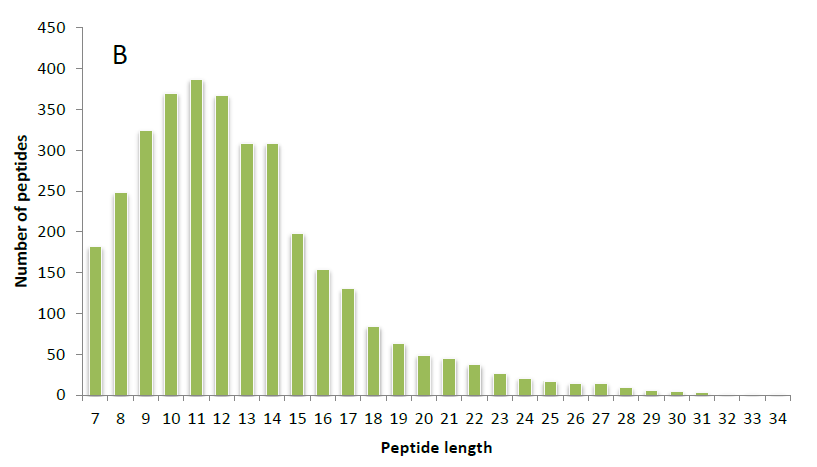

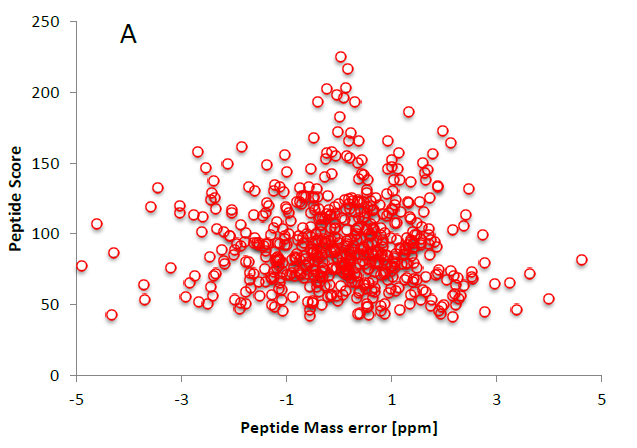

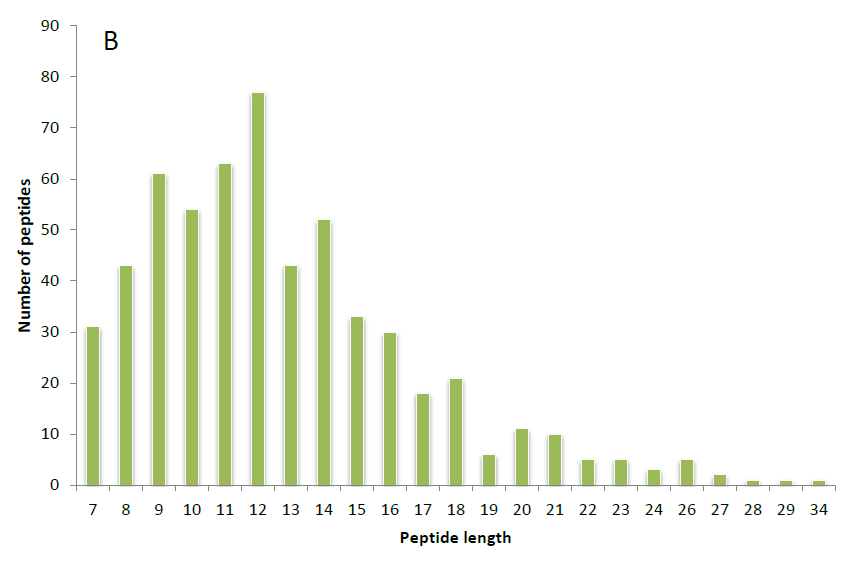


A

B

C

D

**Supplementary figure 1.**

**A,B.** Quality control validation of Acetylation MS data；**C, D.** Quality control validation of Succinylation MS data

1. **B.**


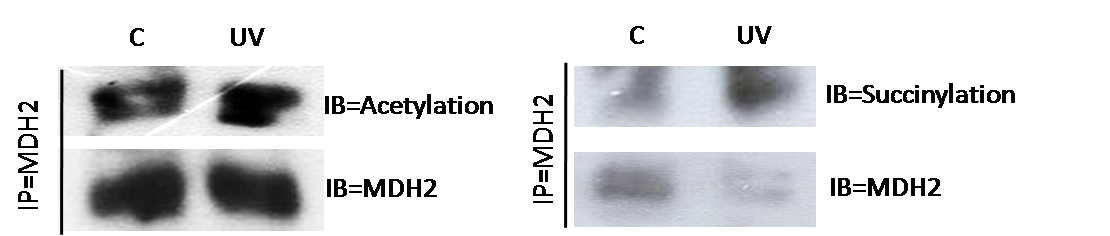


**Supplementary figure 2.** MDH2 acetylation and succinylation level under UV treatment.

1. Acetylation level of MDH2 in HeLa cells before and after UV treatment. HeLa cells were treated with 120J/m^2^ UV radiation and the supernatant of the lysate were collected incubated with anti-MDH2 antibody. The total level of MDH2 and the acetylation level of MDH2 were detected by MDH2 antibody and anti-lysine aceylation antibody. Similar approach was used for succinylation detection in B.


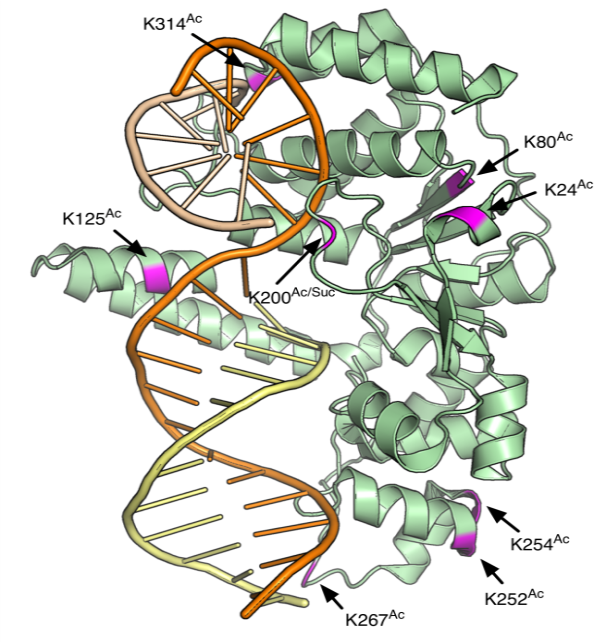


A.


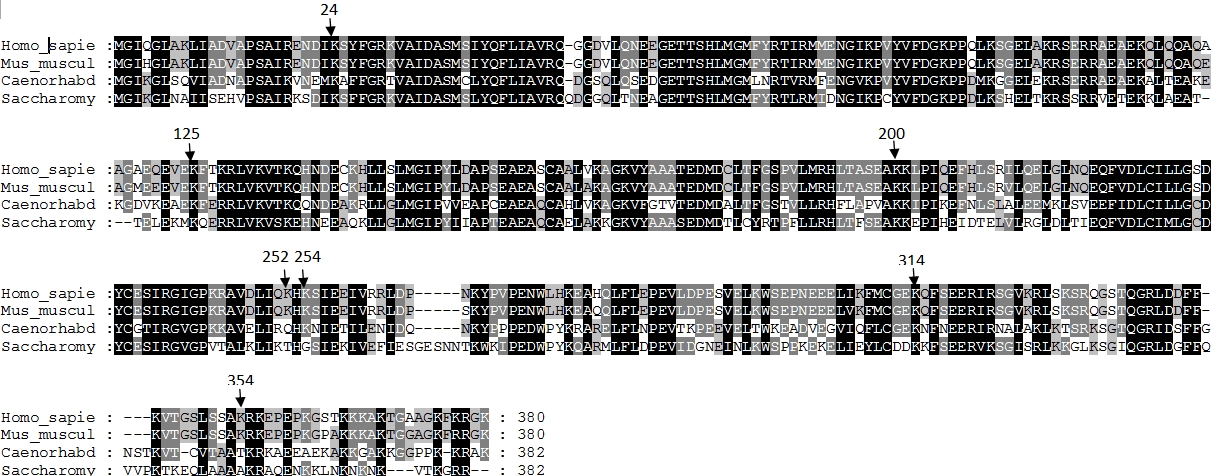


B.

**Supplementary figure 3.**

A. 3D structure view of FEN1 acetylation sites (in aubergine color) based on previous work and this study. B. Clustal W alignment of FEN1 acetylation sites identified in this study. FEN1 homologs from *H. sapiens, M. musculus, Caenorhabd* and *S. cerevisiae* are compared and the acetylation site identified in this study are labeled with arrows.

**
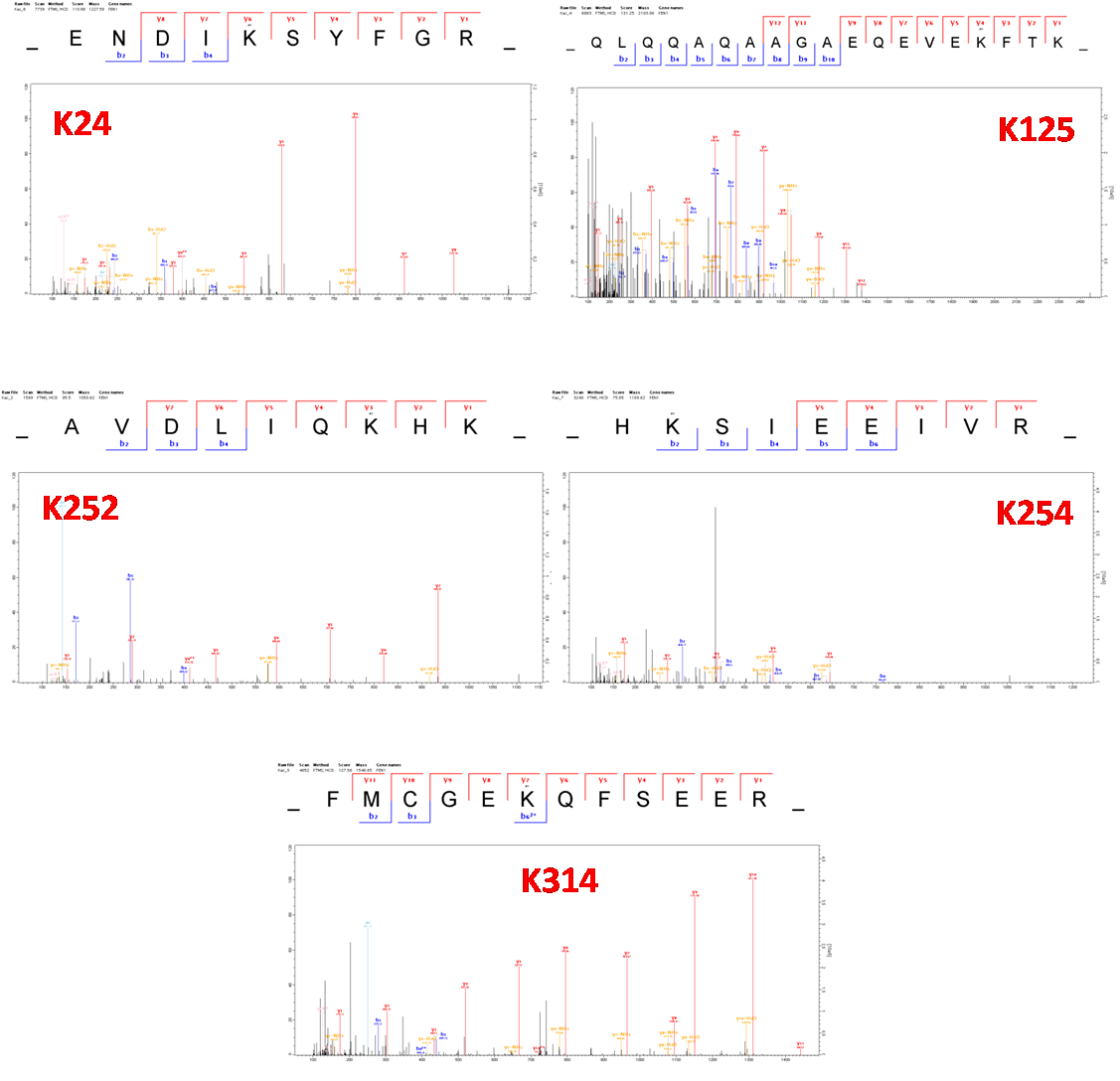
**

**Supplementary figure 4.** MS search results of FEN1 acetylation sites


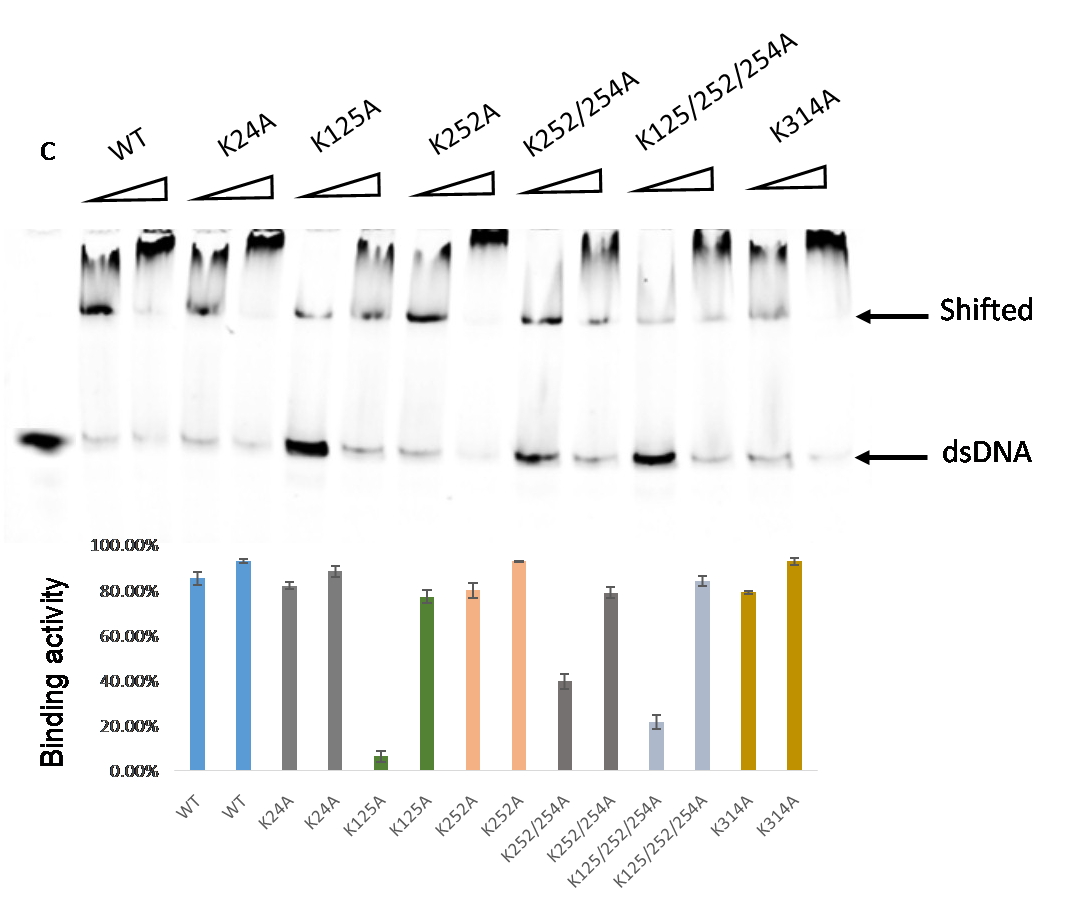


C.


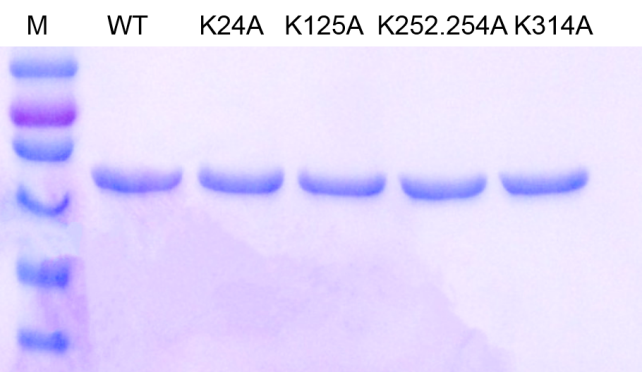


A.

B.

Oligonucleotides sequence：

Oligo1:GATGACGAGCAGTCCTAACTGGAAATCTAGCTCTGTGGAGGAACTCCACAGAGCTAGATTTCCC

Oligo2 : (FAM)-TTTTTAGTTAGGACTGCTCGTCATC

**Supplementary figure 5.**

A. SDS-PAGE of purified FEN1 mutant proteins.

B. Oligonucleotides sequences for DNA binding and activity assays.

C. Quantification of DNA binding activity in different FEN1 proteins by Image J.

**
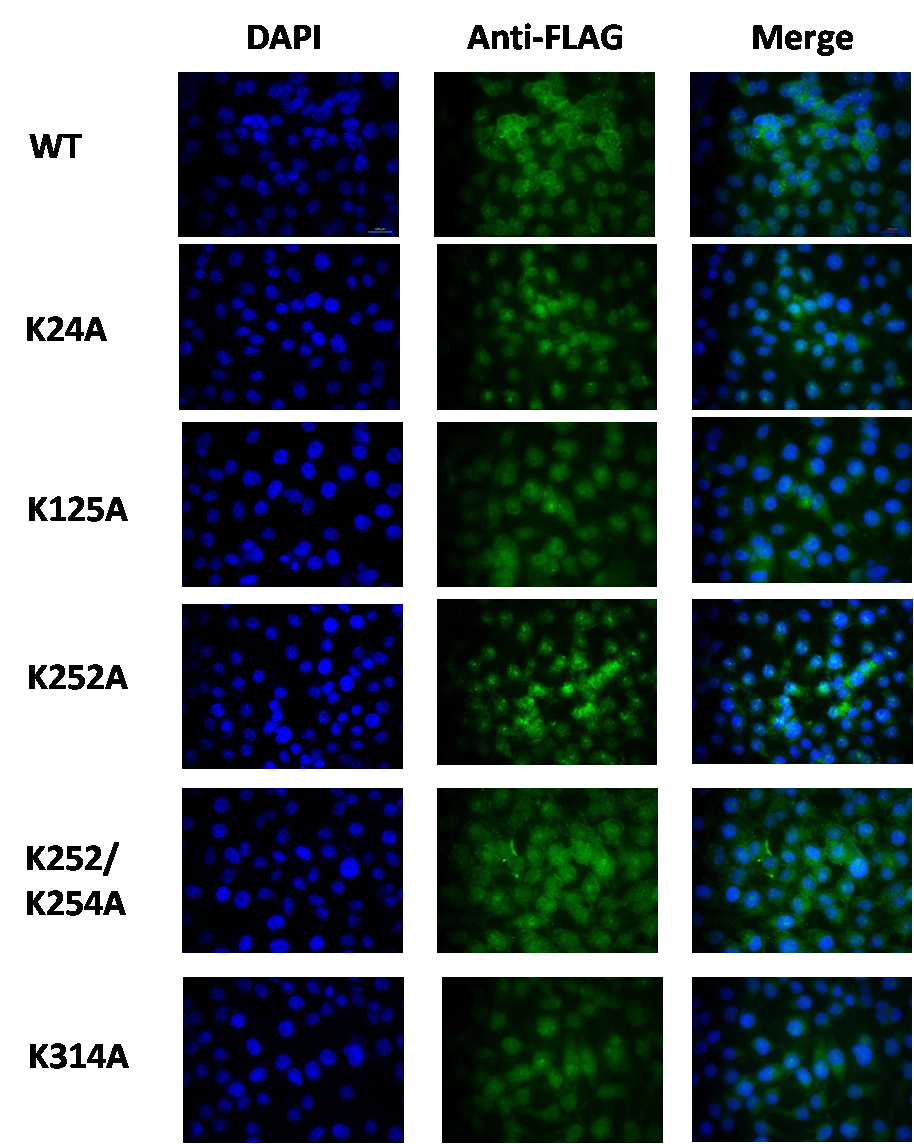
**

**Supplementary figure 6.**

Validation of cell transfection efficiency using immunofluorescence cell staining before FACS. HeLa cells were transfected with FLAG tagged wild-type or mutant FEN1 plasmids and stained sequentially with primary antibody (anti-FLAG) and [Alexa Fluor](http://www.abcam.com/secondary-antibodies/validated-alexa-fluor-secondaries-for-guaranteed-performance) secondary antibody for Immunofluorescence.
